# Molecular Mechanisms Linking Air Pollution-Induced Oxidative Stress to Inflammation and Human Disease

**DOI:** 10.3390/jox16060185

**Published:** 2026-09-26

**Authors:** Kumud Kant Awasthi, Garima Awasthi, Rajbala Verma, Mahipal Singh Sankhla, Pallavi Saxena, Manpreet Kaur, Ruchi Pathania, Harsh Pandey

**Affiliations:** 1 School of Basic & Applied Sciences, K. R. Mangalam University, Gurugram 122103, HR, India; kkantawasthi@gmail.com (K.K.A.); gariimaa21@gmail.com (G.A.); mahi4n6@gmail.com (M.S.S.); 2 Department of Zoology, University of Rajasthan, Jaipur 302004, RJ, India; verma.rajbala@gmail.com; 3 School of Sciences, Woxsen University, Hyderabad 502345, TL, India; 19octpallavi89@gmail.com; 4 School of Pharmaceutical Sciences, CGC University, Mohali 140307, PB, India; mksuprachem@gmail.com; 5 Department of Medicinal Chemistry, University of Florida, Gainesville, FL 32610, USA; 6 Department of Biotechnology and Chemical Engineering, Manipal University Jaipur, Jaipur 303007, RJ, India

**Keywords:** air pollution, oxidative stress, reactive oxygen species, inflammation, molecular mechanisms

## Abstract

Air pollution is a major public health problem worldwide, causing millions of premature deaths every year due to its association with diseases of the respiratory, cardiovascular, neurological, metabolic and carcinogenic systems. A major mechanism of pollutant-induced toxicity has been well identified as oxidative stress. The overproduction of reactive oxygen/nitrogen species causes direct damage to cellular redox homeostasis and activation of inflammatory signaling pathways. This review provides a broad overview of the molecular mechanisms linking ambient air pollutants, such as particulate matter, nitrogen oxides, ozone, sulphur dioxide and volatile organic compounds, with health effects mediated by oxidative stress. Mitochondrial dysfunction, endoplasmic reticulum stress, and epigenetic reprogramming were critically discussed with key signalling pathways such as nuclear factor erythroid 2-related factor 2, nuclear factor kappa B (NF-κB), mitogen-activated protein kinase, and NOD-like receptor protein 3 inflammasome. Tissue-specific effects on respiratory, cardiovascular and nervous systems are also discussed. Susceptible populations and critical exposure windows are identified. Emerging advances in biomarker discovery, and mechanistic in vitro and in vivo approaches, are also summarized as promising tools to improve exposure assessment and disease prediction. Finally, current therapeutic approaches targeting oxidative stress and inflammation, as well as preventive measures to reduce pollutant exposure, are discussed to support precision environmental health and evidence-based air quality management.

## 1. Introduction

Air pollution remains a major global environmental health challenge and contributes substantially to respiratory, cardiovascular, neurological, and metabolic diseases. Oxidative stress and inflammation are central mechanisms linking pollutant exposure with cellular injury and disease development [1].

The World Health Organization identifies air pollution as a major global health risk associated with millions of premature deaths annually (WHO, 2024 [2]). It is also implicated in increased susceptibility to the severe health effects of COVID-19. Pollutants include particulate matter (PM; e.g., soot, dust, and smoke), nitrogen oxides (NOx), sulfur dioxide (SO_2_), carbon monoxide (CO), ozone (O_3_), and volatile organic compounds (VOCs). Characteristic organics found in ambient air include polycyclic aromatic hydrocarbons (PAHs), phenols, and aldehydes. Exposure can occur via inhalation, ingestion, and skin contact [1]. PM is defined as a mixture of solid and/or liquid particles suspended in air, typically subdivided into fine PM (<2.5 μm), coarse PM (2.5–10 μm), and supercoarse PM (>10 μm). Its components include elemental carbon (soot), black carbon (BC), organic carbon (OC), metals (e.g., Pb, Zn, Fe, Cu, Ni, Al, Cr), and mineral components. OC can be further categorized into primary organic aerosol (POA) from the incomplete combustion of fuels and secondary organic aerosol (SOA) formed from gas-phase VOCs via a complex series of physical and chemical processes. The elemental concentration of PM changes spatially and temporally [3].

Oxidative stress plays a major role in air pollution toxicity [1]. Particulate matter can enter the body through multiple routes and mechanically alters cells and tissues [4]. These alterations contribute to the toxic response of pollutants and involve particles, organic constituents, metals, and associated pathways. Long-term exposure to these materials, at subtoxic concentrations, can also provoke toxicity [5]. Air pollution can initiate a cascade of oxidative stress, cellular injury, and inflammatory responses, with reactive oxygen species (ROS) acting as key mediators between pollutant exposure and disease development. Experimental evidence from diverse environmental toxicants demonstrates that oxidative stress can disrupt cellular redox homeostasis, induce DNA damage, and contribute to cytotoxic and tissue-level injury. Silver nanoparticles, for example, have been shown to induce oxidative stress, cytotoxicity, and DNA damage, highlighting the role of ROS-mediated cellular dysfunction in environmental exposure-related toxicity [6]. Similarly, pesticide exposure to bifenthrin and β-cyfluthrin has been associated with ROS-dependent neurotoxicity, genotoxicity, and histopathological alterations [7,8], while β-cyfluthrin-induced oxidative and genotoxic stress can be modulated by antioxidant intervention [9]. Recent evidence further demonstrates oxidative stress and growth inhibition following nanoparticle exposure in plants, supporting the broader relevance of redox imbalance as a mechanism of environmental toxicity [10]. There is a significant contribution of vascular endothelial and epithelial cells to pollutant toxicity, and mucus secretion, barrier infiltration, and intracellular signals from macrophages cannot fully explain the vast toxicity of pollutants. These cells release numerous damaging molecular patterns and signals, such as vascular endothelial growth factor, interleukin 6, heparin-binding epidermal growth factor, DNA, and malondialdehyde low-density lipoprotein complexes [11]. Inhaled PM may exert systemic effects through pulmonary oxidative stress and inflammatory mediators, autonomic nervous system perturbation, and, particularly for ultrafine particles or soluble constituents, possible translocation beyond the lung [12].

The main health outcomes reviewed here are categorized according to their main target systems: respiratory, cardiovascular, neurological, metabolic, reproductive, and cancer. The results are discussed in light of the key ambient pollutants, particulate matter (PM), nitrogen oxides (NOx), ozone (O_3_), sulfur dioxide (SO_2_), carbon monoxide (CO), volatile organic compounds (VOCs) and organic and metal fractions associated with PM. The following sections discuss the oxidative stress, inflammatory, mitochondrial, transcriptional and epigenetic mechanisms by which these pollutants may contribute to the development and progression of disease. Evidence discussed in this review is interpreted according to its source, including human studies, experimental animal models, and in vitro investigations. Mechanistic findings from experimental systems are distinguished from evidence directly demonstrated in human populations [5,12].

### Literature Search and Review Approach

This narrative review summarizes evidence for molecular mechanisms linking air pollution exposure to oxidative-stress, inflammation, and human disease. The relevant literature was identified from major scientific databases using keyword combinations related to air pollution, particulate matter, oxidative stress, reactive oxygen species, inflammation, molecular mechanisms and human health. PubMed/MEDLINE, Scopus, Web of Science, and ScienceDirect databases were searched with major concepts combined using (air pollution OR air pollutants) AND (particulate matter OR PM2.5 OR PM10) AND (oxidative stress OR ROS) AND (inflammation OR inflammatory signaling) AND (molecular mechanisms OR Nrf2 OR NF-κB OR MAPK) AND (human health OR health outcomes OR disease). Further searches were performed using pollutant-specific keywords such as ozone (O_3_), nitrogen oxides (NOx), sulfur dioxide (SO_2_), carbon monoxide (CO), volatile organic compounds (VOCs) and polycyclic aromatic hydrocarbons (PAHs). Eligibility criteria were relevant evidence on pollutant exposure, oxidative stress responses, inflammatory signaling, molecular mechanisms or associated health outcomes. Studies with direct mechanistic relevance to the objectives of this review were prioritized and human, animal, and in vitro studies were considered. Evidence was qualitatively ranked by study design, methodological relevance, directness of evidence, mechanistic strength, consistency of findings and relevance to human health. Preference was given to recent primary studies and authoritative reviews, although landmark studies were retained when they provided important mechanistic evidence. Because of the heterogeneity of study designs, exposure conditions, biological models, endpoints, and outcome measures included in this narrative review, no quantitative weighting of individual studies or statistical meta-analysis was performed.

Evidence was synthesized in a thematic rather than a quantitative way. The synthesis was structured by broad pollutant categories and their linked biological mechanisms, namely: oxidative and redox imbalance, inflammatory signaling, mitochondrial dysfunction, ER stress, epigenetic and transcriptional reprogramming, susceptibility and exposure windows, and tissue- and organ-level outcomes. Findings from animal and in vitro models were separated from evidence from human studies, and mechanistic findings from experimental systems were interpreted separately from evidence demonstrated directly in human populations.

## 2. Reactive Oxygen and Redox Imbalance

Oxidative stress is defined as the imbalance of pro-oxidants and antioxidants and the consequent damage to cells and their components [13,14,15]. As shown in Figure 1, exposure to air pollution is now viewed as a risk factor for various chronic diseases; the key mechanism of action appears to be oxidative stress, initiated by the generation of reactive oxygen and nitrogen species (ROS/RNS) [16]. The generation of ROS/RNS is primarily influenced by external environmental factors and can originate either directly or indirectly, or there may be an equilibrium effect involving both direct and indirect production of reactive oxygen and nitrogen species (RONS). RONS generation and redox imbalance are pivotal in air pollution-induced oxidative stress pathways [16,17,18,19]. Following exposure to ambient pollutants, cellular levels of RONS are altered, and the consequent imbalance overwhelms the cell’s antioxidant defenses [16]. The cell strives to restore redox homeostasis, primarily through a transcriptional response governed by nuclear factor E2-related factor 2 (Nrf2), which regulates the expression of more than 100 genes encoding antioxidant and detoxifying proteins [20]. However, exposure to pollutants that directly induce or amplify RONS generation can lead to keratinocyte depletion of the principal antioxidant glutathione (GSH) and impair antioxidant defenses, including glutathione-dependent and enzymatic antioxidant systems [21]. Consequently, air pollution modulates the system in ways that deviate from the prototypical Nrf2-centered response [20].

High concentrations of GSH prevent dipalmitoylphosphatidylcholine (DPPC)-lipid peroxide transformation and accumulation and thereby various downstream oxidations [22]. Decrement of GSH and/or damage to GSH redox regulators fuels by the pollutants lead to premature DPPC-peroxide formation. The GSH prevention mechanism and the relevant Nrf2-uncoupling pathway offer new insight into pollutant oxidative stress mechanisms [20,22]. Moderate oxidative stress may activate Nrf2-mediated cytoprotective responses, whereas persistent or excessive oxidative burden can overwhelm antioxidant defenses and promote inflammatory signaling and cellular injury.

The airways, being in direct contact with pollutants, are an early target. Within minutes of exposure to concentrated particulate matters, human primary bronchial epithelial cells activated interleukin-6 (IL-6) and regulated upon activation normal T-cell expressed and secreted (RANTES) production [23,24]. Lipid peroxidation triggered by PM490 exposure correlated with IL-6 and RANTES production [25]. A rise in intracellular hydrogen peroxide (H_2_O_2_) occurred shortly after exposure to concentrated PM permitting investigation of H_2_O_2_ as a second-messenger species responsible for the early inflammatory response [1]. The biological effects of PM are influenced not only by particle size but also by chemical composition. Transition metals such as iron, copper, nickel, chromium, and other redox-active components can participate in reactions that generate reactive oxygen species and promote oxidative damage. Organic constituents, including polycyclic aromatic hydrocarbons (PAHs), aldehydes, and other combustion-derived compounds, can activate cellular stress and inflammatory pathways. Some organic compounds can also interact with aryl hydrocarbon receptor-dependent signaling and alter gene expression. Thus, PM toxicity reflects the combined effects of particle characteristics and associated chemical constituents rather than particle mass alone. Table 1 summarizes the major oxidative stress and inflammatory biomarkers associated with air pollution exposure. These biomarkers reflect key molecular events triggered by pollutants, including excessive ROS/RNS generation, depletion of antioxidant defenses, activation of redox-sensitive signaling pathways, inflammatory cytokine release, mitochondrial dysfunction, and endoplasmic reticulum stress. Collectively, they serve as valuable indicators for assessing pollutant-induced cellular injury, disease progression, and potential therapeutic responses in environmental health studies.

The oxidative stress caused by air pollution is determined by the concentration of pollutants, the exposure duration, the chemical composition and the individual susceptibility. At lower or transient exposure levels, cellular antioxidant systems such as Nrf2-regulated responses and glutathione-dependent defenses may be able to partially maintain redox homeostasis. At higher concentrations or with longer exposure, the production of reactive oxygen and nitrogen species may exceed antioxidant capacity, resulting in GSH depletion, lipid and protein oxidation, DNA damage, mitochondrial dysfunction, and activation of inflammatory pathways. Thus, the relationship between pollutant concentration and oxidative stress is not necessarily linear and can be influenced by the pollutant mixture, the composition of the particles, the duration of exposure, and the biological system investigated. The oxidative potential of particulate matter varies depending on its content of transition metals, organic compounds and other redox-active components. Therefore, in addition to the duration of exposure, concentration–response relationships must be considered considering pollutant composition, deposited dose, and the oxidative stress endpoint measured. Alterations in various molecular and cellular markers reflect oxidative stress and inflammatory responses to air pollution. However, these markers are markers of different biological processes and should not be interpreted as equivalent indicators. ROS and RNS are markers of a redox imbalance, while GSH depletion is a marker of impaired antioxidant defense. HO-1 and Nrf2 are largely protective antioxidant responses, while MDA, protein carbonyls and 8-OHdG are markers of oxidative damage to lipids, proteins and DNA respectively. Activation of inflammatory signaling is indicated by inflammatory mediators such as IL-1β, IL-6, IL-18 and TNF-α. Hence, the biological role and context of exposure, as well as available experimental or human evidence, should be considered for the interpretation of each marker (Table 2).

## 3. Inflammatory Pathways Activated by Pollutant-Driven Oxidative Stress

Air pollutants elevate the generation of reactive oxygen species and nitrogen species, which overwhelm cellular antioxidant defenses and cause oxidative alterations ranging from reversible redox signaling to irreversible macromolecular damage. Various pathways driven by this oxidative stress subsequently promote inflammation in multiple tissues and initiate systemic exposure–response relationships [34].

Oxidative stress, a disturbance between the formation and elimination of reactive species, is a condition in which pro-oxidants overwhelm the endogenous antioxidant defense system. This imbalance induces oxidative damage to macromolecules such as lipids, proteins, and DNA, causing alterations in biochemical and physiological processes. Polyaromatic hydrocarbon subcomponents of particulate matter activate oxidative stress pathways through the aryl hydrocarbon receptor [1]. In addition to direct macromolecular damage, pollutant-induced oxidative stress triggers inflammatory response pathways as shown in Figure 2. The nuclear factor kappa B (NF-κB) signaling pathway enhances the transcription of proinflammatory cytokines such as interleukin (IL)-1β, IL-6, and tumor necrosis factor-α, whereas mitogen-activated protein kinase (MAPK) cascades lead to the expression of cyclooxygenase-2 and additional proinflammatory mediators [35]. Furthermore, oxidative stress signals regulate the NLRP3 inflammasome, motivating the proteolytic maturation and extracellular release of proinflammatory cytokines like IL-1β and IL-18 [36]. 

### 3.1. Nuclear Factor Kappa B Signaling

Oxidative stress can activate NF-κB signaling through IκB phosphorylation and degradation, followed by NF-κB nuclear translocation and transcription of inflammatory genes [3]. Pollutants can provoke the formation of ROS in mitochondria, leading to oxidative impairment of lipids, proteins, and DNA. This oxidative burden activates signaling systems that initiate the transcription of proinflammatory cytokines involved in tissue inflammation and repair. NF-κB assembly and activity are tightly regulated by redox status. ROS also promote the aggregation/depolymerization of actin and contribute to chromatin remodeling. Ionizing radiation, ultraviolet light, oxidants, and reactive species generate DNA damage, another signal for NF-κB pathway stimulation, contributing to global genetic reprogramming [37]. Pollutant-induced mitochondrial and nuclear DNA damage may generate cytosolic DNA signals that activate the cGAS-STING pathway and amplify inflammatory responses. Cytosolic DNA and certain types of DNA lesions stimulate the cGMP AMP synthase (cGAS) stimulator of interferon genes (STING) signaling and induce NF-κB activation. A feedback loop that amplifies proinflammatory signaling can be established when NF-κB induces the transcription of genome regulators that incite further NF-κB activation [38].

### 3.2. Mitogen-Activated Protein Kinase Cascades

Air-pollutant exposure can stimulate a plethora of biological responses by engaging with innate immune receptors such as Toll-like receptors and the receptor for advanced glycation end-products, which in turn manage various downstream signaling pathways involved in the mediation of inflammatory responses, thereby establishing a link to the proinflammatory and pro-angiogenic nature of the components of airborne particulate matter [1]. The activation of different sub-families of mitogen-activated protein kinases (MAPKs) such as c-Jun N-terminal kinases (JNKs), extracellular-regulated kinases (ERKs), and p38 MAPKs has also been shown to play a pivotal role in orchestrating the biological effects induced by oxidative stress [39]. MAPKs can be categorized into three major classes: JNK, ERK, and p38, based on the degree of homology of their N-terminal and C-terminal activation domains. In mammalian cells, the MAPK pathway is activated by a variety of extra- and intracellular stimuli, such as cellular stresses. The activation often occurs in a so-called “p38-MAPK-JNK-c-Jun activator protein-1 (AP-1)” signaling module. It operates in parallel with a “p38-MAPK-ERK-c-Fos-AP-1” module, thereby leading to the induction of specific event(s), such as stress-response genes, according to the nature and/or amplitude of the stimulus [40]. The mammalian JNKs activate the c-Jun transcription factor, while the membrane-bound ERKs regulate the activities of the Ets and c-Myc transcription factors. The c-Jun, Ets, and c-Myc proto-oncogene-laying-down transcription factors are involved in the transduction of various stress signals arising from, among others, atmospheric disturbance and PM exposure, and ultimately lead to the expression of genes involved in stress adaptation and defense [41].

### 3.3. NLRP3 Inflammasome and Cytokine Maturation

Airborne pollutants exert harmful effects on the respiratory tract and other organ systems, aggravating diseases such as asthma, pulmonary infections, chronic obstructive pulmonary disease, carcinogenesis, atherosclerosis, myocardial infarction, stroke, neurodegeneration, and more [1]. Toxicological investigations have identified oxidative stress as a central mechanism mediating these adverse effects. Oxidative stress refers to an imbalance between the generation of reactive oxygen and nitrogen species (RONS) and the ability of biological systems to eliminate them, resulting in tissue damage. Increasing evidence indicates that pollutants induce oxidative stress in the lung or the bloodstream, activating damaging signaling cascades in distant organs. Phagocytosis of particles and bacteria by resident macrophages, epithelial uptake of gases like nitrogen dioxide and ozone, and circulatory delivery of soluble gases such as carbon monoxide generate RONS, leading to redox imbalance and triggering downstream signaling. Such events set the stage for upregulation of proinflammatory genes under the regulation of key transcription factors, including nuclear factor kappa B (NF-κB), mitogen-activated protein (MAP) kinases, and nuclear factor erythroid 2-related factor 2 (Nrf2) [42,43].

## 4. Molecular Crosstalk Between Oxidative Stress and Inflammation

Oxidative stress caused by air pollution can also change cell metabolism, which is an important connection between molecular stress responses and the development of diseases. Mitochondrial dysfunction may result in impaired oxidative phosphorylation and ATP synthesis associated with increased electron leakage and reactive oxygen species production. Persistent oxidative stress can result in alterations of glucose and lipid metabolisms, mitochondrial energy homeostasis and redox-dependent metabolisms. Additionally, inflammatory signaling can modulate cellular metabolism by increasing metabolic demand and altering substrate utilization. These interrelated changes may lead to endothelial dysfunction, insulin resistance, metabolic derangements and tissue injury. Thus, metabolic dysregulation represents another mechanistic link between pollutant exposure, oxidative stress, inflammation and chronic disease. Air pollution-induced oxidative stress promotes inflammation and consequent pathologies of the cardiovascular, respiratory, and nervous systems [1]. Cytokine signaling may trigger detrimental neurovascular and neurodegenerative events in the brain. Multiple pathways profit from oxidant emission to activate inflammatory mediators [44], including nuclear factor kappa B (NF-κB) and mitogen-activated protein kinases (MAPK), with transient release of damage-associated molecular patterns [45]. Pollutants, and particles (PM) in particular, instigate significant perturbance of cellular antioxidant systems, resulting in global redox imbalance and the elevation of reactive oxygen species (ROS) or state of oxidized glutathione (GSSG) [46]. Pollutant-driven ROS and organelle-specific, cell-intrinsic stresses instigate mitochondrial injury characterized by the excretion of mitochondrial DNA and oxidized ATP, and trigger elaborate, auto-amplifying stimuli at the cellular level. Pollutant-induced oxidative stress can impair mitochondrial function and activate endoplasmic reticulum stress responses, thereby promoting inflammatory signaling and cellular injury. A second branch of ER stimuli relay protective signaling throughout the network, prior to acquiring further pollutants [47].

### 4.1. Mitochondrial Dysfunction and Damaging Feedback Loops

The general effects of oxidants lead to mitochondrial dysfunction, which can create a damaging feedback loop. Disrupting the inner membrane potential opens the mitochondrial permeability transition pore (mPTP), leading to the release of pro-apoptotic factors and amplification of cytosolic oxidative stress [1]. Mitochondrial fission generates fragmented mitochondria that are unable to maintain effective bioenergetics, whereas excessive fusion prevents elimination of damaged organelles [27]. Alterations in mitochondrial fission, fusion, and mitophagy can contribute to mitochondrial dysfunction, ROS generation, and cellular injury, although these responses may vary across pollutant types and experimental models [3].

### 4.2. Endoplasmic Reticulum Stress and the Unfolded Protein Response

Accumulation of unfolded proteins in the endoplasmic reticulum (ER) leads to disruption of both cellular and tissue homeostasis and activates the unfolded protein response (UPR), the transcriptional program that through a triage-like mechanism induces the expression of chaperones, protein synthesis inhibition factors, and proteases, and ultimately initiates apoptosis if damage is irreparable [48]. The UPR is regulated by three ER stress sensors in mammals: inositol-requiring protein 1 (IRE1), activating transcription factor 6 (ATF6), and protein kinase RNA-like ER kinase (PERK) [49]. Numerous exogenous signals, including cigarette smoke, carbon black, and fine particulate matter, have been shown to induce ER stress in the lung. Ambient concentrations of particulate matter induce UPR elements such as ATF4, spliced XBP1, and GRP78, and subsequent apoptosis in both lung and liver tissues; these effects can be abrogated by both atmospheric filtration and prohibition of indoor smoking [50]. IRE1 is a well-known regulator of inflammation and apoptosis during chronic ER stress and has also been implicated in short-term responses. NF-κB activation, transcriptional induction of proinflammatory factors, and apoptosis all have been shown to depend on IRE1. Cigarette smoke extract activates the NF-κB and mitogen-activated protein kinase pathways through IRE1 in lung epithelial cells, and particulate matter-mediated UPR elements are connected to NF-κB induction [1]. ER stress has been causally linked to a broad range of lung diseases, including chronic obstructive pulmonary disease, asthma, and pulmonary hypertension [51].

## 5. Tissue and Organ-Level Consequences

Air pollution contributes to adverse respiratory, cardiovascular, neurological, reproductive, and carcinogenic outcomes. These effects are mediated through multiple mechanisms, including oxidative stress, inflammation, vascular dysfunction, and organelle injury [1,52].

### 5.1. Cardiovascular System

Cardiovascular causes account for more than two-thirds of the mortality associated with air pollution [34]. Oxidative stress is an important pathway mediating air pollution’s effects on the cardiovascular system. Pollutants such as particulate matter and gases affect oxidant/antioxidant balance within cardiomyocytes, vascular cells, platelets, and leukocytes. This response alters physiological functions and contributes to disturbances in blood flow, cardiac output, cardiac rhythm, and hemostasis [1].

### 5.2. Respiratory System

Patients with chronic respiratory diseases are especially susceptible to ambient airborne pollutants because of their greater exposure and the significant oxidative stress these components cause to the tissues of the respiratory tract upon inhalation [18]; therefore, large quantities of free radicals originate from the membrane of respiratory epithelial cells, substantially increasing the local burden of free oxidative species [1]. These events lead, in turn, to the oxidation of cellular constituents such as proteins, nucleic acids, and membrane lipids. The interaction of airborne pollutants with other tissue components produces new reactive oxygen species (ROS) directly within the plasma membrane and provides an indirect pathway for the generation of more ROS. Moreover, several intermediary components produced from the oxidative alteration of lipids, proteins, and nucleic acids can induce further damage to the cells and surrounding matrix, as well as propagate systemic responses from extra-pulmonary organs.

### 5.3. Neurovascular and Neurodegenerative Implications

Exposure to air pollutants has been linked to inflammation and a higher risk of neurodegenerative diseases. Central nervous system components, particularly blood–brain barrier cells such as astrocytes and brain endothelial cells, are directly affected by ambient particulate matter exposure [27]. Phagocytosis of particulate components leads to oxidative stress (alters redox balance and promotes reactive oxygen species formation) and induces inflammatory mediators. Pollutant penetration into the brain parenchyma triggers microglial immune responses that exacerbate damage [53].

Ambient particulate matter (PM) exposure is associated with microglial and astrocytic activation, neuroinflammation, and cognitive dysfunction. Significant associations between psychotic experiences and PM10 exposure are observed, independent of socioeconomic, marital, drug use, and generalized anxiety disorder factors. Mitochondrial damage and allostatic load (the cumulative biological burden incurred through exposure to chronic stressors) also show a clear association with brain health [54]. Chronic exposure to environmental stressors may increase allostatic load and contribute to adverse neurological outcomes [55]. Particulate matter, mercury, volatile organic compounds, ozone, carbon monoxide, and nitrogen dioxide exposure during the prenatal period impairs cognitive development and leads to neurodegenerative diseases [56].

## 6. Epigenetic and Transcriptional Reprogramming in Response to Pollutants

Air pollutant exposure can induce acute transcriptional responses and, in some experimental and human studies, persistent epigenetic alterations [57]. These effects may involve DNA methylation, histone modifications, chromatin accessibility, and non-coding RNA regulation, although their persistence and functional consequences remain incompletely understood. Transcription factors, chromatin-modifying enzymes, and other effectors that orchestrate the early transcriptional response are retained on the genome, where they influence the activity of distal genomic sites to shape the amplitude and temporal dynamics of transcriptional reprogramming (Figure 3).

Transcriptional and epigenetic reprogramming of resident cells by environmental pollutants shapes repeated transcriptional responses upon subsequent exposures. Pollutants such as ozone, particulate matter, diesel exhaust, and others drive these distinct patterns of gene expression following separate exposures [58,59]. Other stressors, including proteotoxic damage, DNA damage, and metabolic imbalance similarly generate unique and specific gene expression profiles [60,61].

## 7. Susceptibility, Exposure Windows, and Population Health Considerations

Oxidative stress, generated by multiple pollutants through diverse pathways, initiates inflammatory signaling driving a spectrum of pathologies, yet the contribution to human disease risk, susceptible subpopulations, critical exposure windows, and health inequalities remain incompletely understood. Susceptibility encompasses numerous interconnected biological attributes, shaped by genetics, the exposome, and life-history stages such as gestation and childhood, when epigenetic reprogramming, development, and organ maturation occur [51]. Preclinical evidence demonstrates that cardiovascular effects of particulate matter are markedly intensified following fetal exposure and at distinct postnatal periods [62]. Evidence of heightened liver sensitivity exists for pollution-related chemicals and particulate matter at various life stages [63]. In the context of multiple exposures along complex and overlapping pathways, further work is needed to quantify the design and implementation of mitigation strategies that promote health equity and benefit the largest number of individuals throughout life. Susceptible populations include pregnant individuals, fetuses, infants, children, older adults, and individuals with respiratory, cardiovascular, or metabolic disorders. Genetic variation, impaired antioxidant capacity, occupational exposure, and socioeconomic disadvantage may further modify vulnerability. Importantly, increased biological susceptibility and increased exposure should be considered separately.

## 8. Methodological Approaches in Mechanistic Research

Exposure to air pollutants can induce oxidative stress in different cells and tissues, augmenting the risk of cardiovascular and cerebrovascular diseases, asthma, chronic obstructive pulmonary disease, and neurodegeneration. Pollutants within particulate matter (PM), micro- and nanoparticles, gases, and vapors facilitate the production of reactive oxygen and nitrogen species (RONS), compromising the antioxidant defenses of target cells and mounting damaging redox imbalances. Consequently, the initiation and propagation of oxidative stress and inflammation are coupled, forming positive feedback loops that exacerbate the toxic burden. For example, mitochondrial dysfunction and the consequent leak of pro-oxidants perpetuate oxidative stress, while endoplasmic reticulum stress promotes the switch between antioxidant and oxidant enzymes, fueling redox disruption and inflammation. Redox imbalance and inflammation not only engage one another but also represent key determinants in other mechanistic (epigenetic/transcriptional reprogramming; susceptibility/exposure windows) and organ/tissue (cardiovascular; respiratory; neurovascular/neurodegenerative) dimensions; hence, dissecting these interactions at the molecular, cellular, and organ levels is essential to elucidate air pollution mechanisms and devise therapeutic strategies [1].

Environmental pollution is a global concern, causing major health problems and premature deaths [64]. Inhalation of airborne particulate matter (PM) and gaseous pollutants is linked to cardiovascular, respiratory, and central nervous system diseases, as well as preterm birth and low birth weight [65,66]. Outdoor air pollution and particulate matter in outdoor air pollution have been classified by IARC as carcinogenic to humans (Group 1). This classification should not be extended to NOx or O_3_ as individual Group 1 carcinogens. Mechanistic understanding of pollutant-induced oxidative stress is aided by in vitro and in vivo studies. Pollutants enter the body through inhalation, skin contacts, and ingestion. Once in the body, pollutants exert potent oxidative effects through formation of reactive oxygen species (ROS) and depletion of antioxidants. Pollutant exposure induces mitochondrial dysfunction, endoplasmic reticulum stress, inflammatory response, and vascular toxicity [1]. Even ambient pollutants such as PM10, PM2.5, NOx, and O3 that meet standard levels may contribute to toxic effects and cause oxidative stress.

Research on air pollution offers critical insights into the relationship between environmental insults and disease, facilitating biomarker discovery and enhancing translational applications. Certain biomarkers may illuminate the mechanistic pathways linking air pollutants and particulates to human health, thereby informing mitigation approaches. Elevated levels of proinflammatory cytokines following acute exposure to urban particulate matters, ozone, and nitrogen dioxide indicate a potential immune response associated with pollution exposure. Alveolar macrophages and pulmonary epithelial cells exposed to particulate matters produce proinflammatory cytokines/cytokine receptors and nitric oxide [67]. Particulate matter stimulates the NOD-like receptor protein 3 (NLRP3) inflammasome in both natural and transformed human airway epithelial cells [68]. Biomarkers reflecting oxidative stress help delineate the mechanistic roots of air pollution health effects. Substantial progress has been made with regard to the understanding of the transcriptional programs activated by air pollutant-induced oxidative stress. Indeed, sensing and transducing oxidative stress signals into adaptive transcriptional responses regulates the cellular adaptations to environmental stressors, such as heavy metals, UV radiation, chemicals, and air pollution [19]. Among the many stress-inducible genes, heme oxygenase-1 (HO-1) has emerged as a critical component of the oxidative stress response and an important biomarker for oxidative stress associated with air pollution per se, as well as the physiological and toxicological responses to other environmental stressors. A major limitation of mechanistic studies is the difference between experimental and real-world exposure conditions. Ambient air pollution represents a complex mixture rather than isolated pollutants. Future studies should increasingly consider source-specific mixtures, co-exposures, and interactions between chemical, environmental, and host-related factors.

Recent studies provide further evidence to support the role of oxidative stress and redox-sensitive ion channels in mediating the biological effects of traffic-related air pollutants. NO_2_ exposure has been linked to airway inflammation and chronic respiratory outcomes, with reactive oxygen species as important mediators of cellular stress. Some TRP channels, including TRPV1, TRPV4, TRPA1 and TRPM2, may contribute to pollutant effects on the airways by sensing oxidative and chemical stress and modulating calcium-dependent cell signaling. Activation of these channels can modulate epithelial responses, the release of inflammatory mediators, oxidative stress and airway reactivity. These mechanisms may provide a potential molecular link between exposure to traffic-related air pollution and chronic respiratory diseases such as allergic asthma. Emerging evidence also suggests that oxidative stress, inflammation and altered cellular signaling may play a role in metabolic outcomes, including diabetes, following exposure to traffic-related pollutants. These results further support the concept that pollutant-induced oxidative stress is associated with disease through multiple, interacting cellular pathways rather than a single molecular mechanism. Experimental studies further suggest that combined exposure to NO_2_ and extreme heat can exacerbate diabetic nephropathy and accelerate liver disease progression in type II diabetes models. These findings highlight the role of oxidative stress and inflammation as important mechanisms linking NO_2_ exposure and environmental co-stressors with respiratory and metabolic disease outcomes [69,70,71,72].

## 9. Potential Therapeutic Targets and Preventive Strategies

Reducing pollutant exposure remains the primary strategy for preventing air pollution-related health effects. Molecular interventions targeting oxidative stress and inflammation remain largely experimental and require further clinical validation. Both therapeutic interventions and preventive measures hold the potential to diminish the harmful health impacts of both fine and coarse air pollution PM and other oxidants. Protection from ambient particulate matter air pollution is significant for averting cardiovascular, metabolic syndrome, and type-2 diabetes disorders [1]. Enabling the antioxidative properties of high-density lipoproteins (HDLs) and the ATP-binding cassette transporter G-1 (ABCG1) helps prevent PM-induced cardiac oxidative stress and cell dysfunction [73,74]. Exposure to ambient PM impairs endothelial progenitor cell functionality through oxidative stress and promotes vascular insulin resistance by rapid hypothalamic proinflammatory cytokine release that results in elevated blood pressure and increased type-2 diabetes risk [75]. Pollutant-induced lung oxidative stress activates Nrf2-mediated antioxidant cellular defenses and cytosolic glutathione transferase despite abundant cytosolic free iron availability and xanthine oxidase induction [76].

Therefore, targeting oxidative stress responses with Nrf2-induced transcriptional detoxifying network upregulation and xanthine oxidase activity downregulation represents a potential mechanistic target that requires further validation in preclinical and human studies. Enforcing urban-planning strategies, substituting clean energy for solid fuels in household cooking and heating, implementing open burning prohibitions, and refraining from smoking tobacco can significantly minimize inhabitants’ exposure to outdoor and indoor pollution, respectively. Besides controlling ambient PM levels, modulating oxidative stress and inflammatory responses constitutes a vital preventive and therapeutic measure for severe cardiovascular, metabolic, and air pollution-associated diseases [51]. Antioxidant interventions should be interpreted cautiously because ROS also participate in physiological signaling. Their effectiveness may depend on dose, timing, tissue specificity, and the balance between protective redox signaling and oxidative damage.

## 10. Conclusion

Air pollution initiates oxidative and inflammatory responses through several interrelated pathways that underlie respiratory, cardiovascular, neurological, and other adverse health effects. Current evidence suggests important roles for pollutant composition, oxidative potential, mitochondrial dysfunction, inflammatory signaling, and host susceptibility. However, there are still major uncertainties about which PM components are most harmful, how mechanistic biomarkers translate to human disease risk, and the effects of chronic low-dose mixtures of pollutants. Future research should focus on environmentally realistic exposure models, better validation of human biomarkers, and the identification of molecular pathways that may be reliable targets for prevention or therapy.

## Figures and Tables

**Figure 1:**
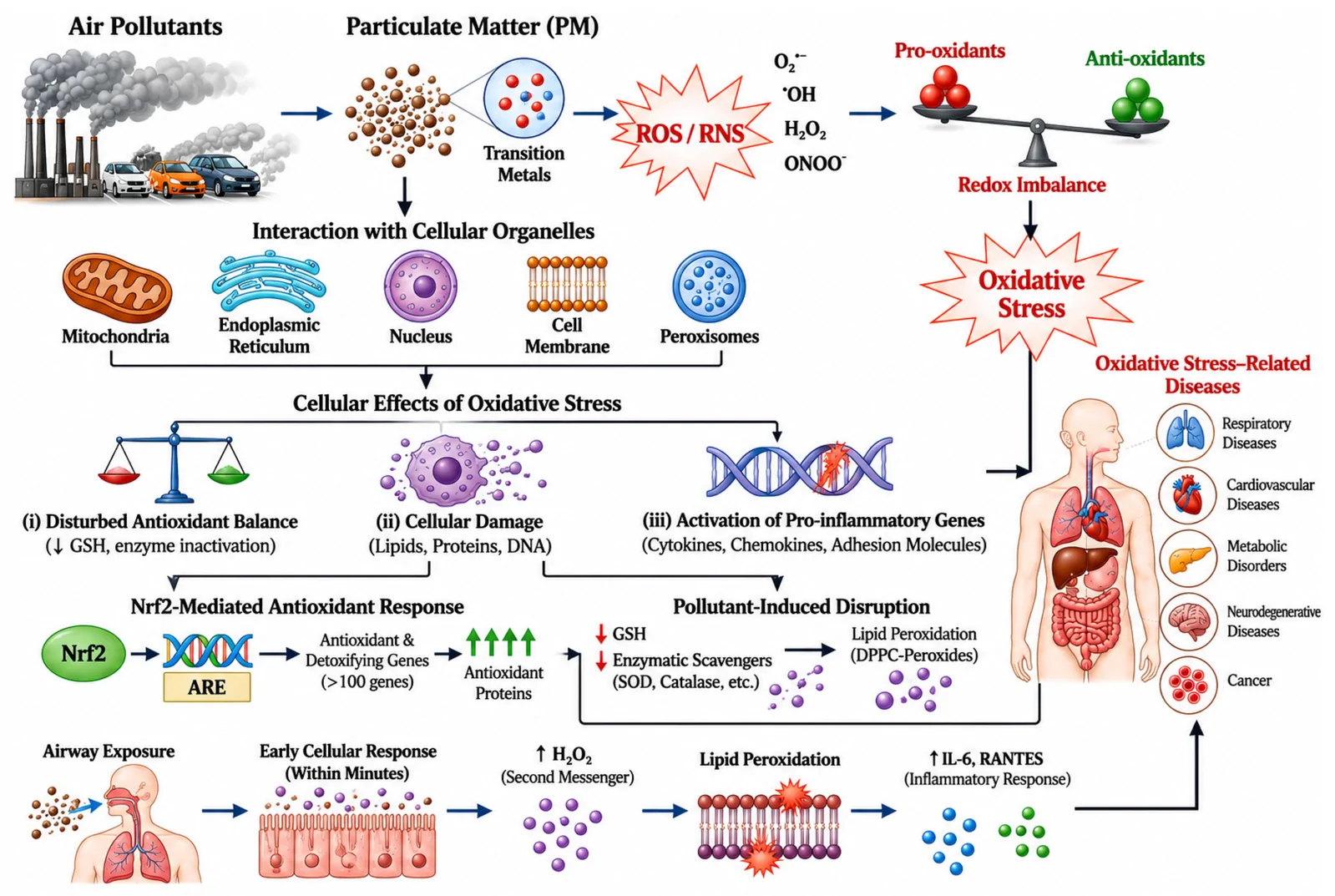
Proposed mechanistic framework of air pollutant-induced oxidative stress. Pollutant exposure may disrupt redox homeostasis and activate interacting oxidative, inflammatory, mitochondrial, and antioxidant responses. Nrf2-mediated antioxidant signaling may initially be adaptive but can become insufficient during persistent oxidative stress. FigureLabs (2026) was used for preparing the figure.

**Figure 2:**
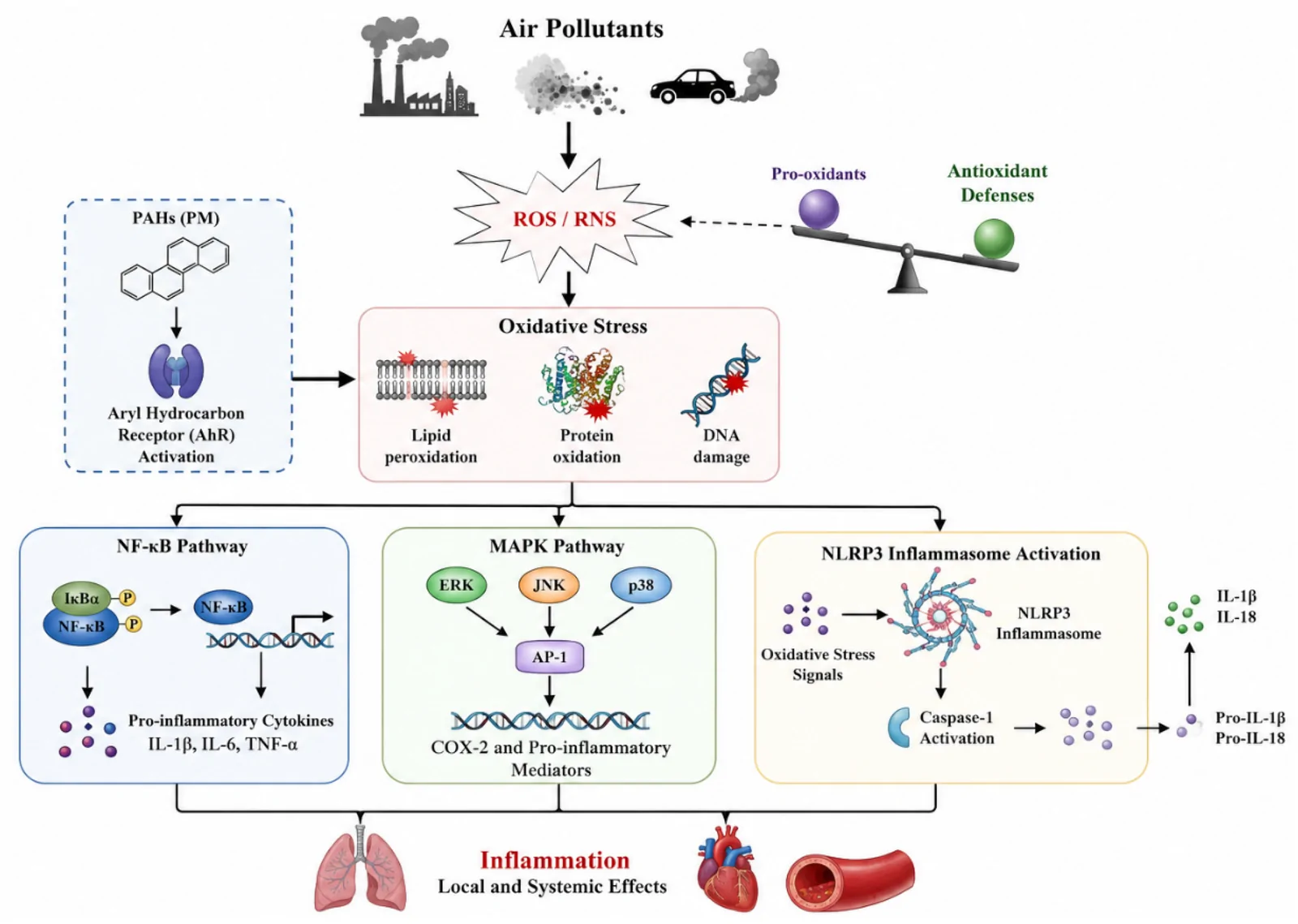
The pathways shown interact with Nrf2-mediated antioxidant defense, which may counteract oxidative injury under moderate stress but become insufficient during sustained exposure. FigureLabs (2026) was used for preparing the figure.

**Figure 3:**
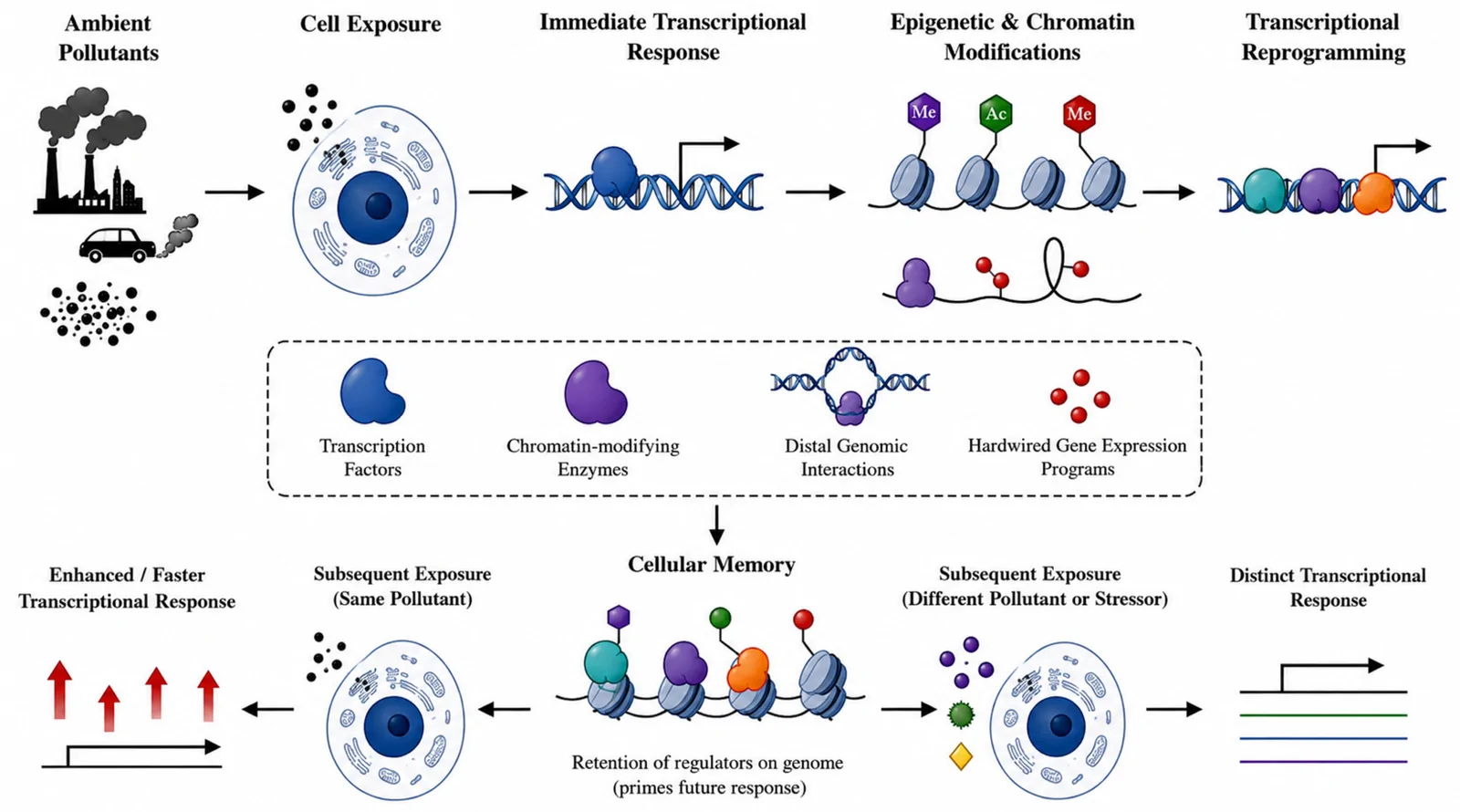
Proposed framework of air pollutant-associated transcriptional and epigenetic alterations. The magnitude, persistence, and biological consequences of these responses may vary according to pollutant type, tissue, exposure duration, and biological context. FigureLabs (2026) was used for preparing the figure.

**Table 1:** Oxidative stress and inflammatory biomarkers associated with air pollution exposure [1,3,26,27].

Biomarker	Category	Change Following Air Pollution Exposure	Biological Significance	Major Pollutants	Clinical/ Experimental Relevance
ROS (Reactive Oxygen Species)	Oxidative stress	Increased	Initiates oxidative stress, lipid/protein/DNA damage	PM2.5, PM10, O_3_, NO_2_, PAHs	Primary indicator of oxidative injury
RNS (Reactive Nitrogen Species)	Oxidative stress	Increased	Nitrosative stress and redox imbalance	PM, NOx	Cellular injury and inflammation
H_2_O_2_	ROS	Increased	Secondary messenger promoting inflammation	PM	Early inflammatory signaling
Glutathione (GSH)	Antioxidant	Decreased	Loss of antioxidant defense	PM, O_3_	Marker of antioxidant depletion
HO-1 (Heme Oxygenase-1)	Antioxidant response	Increased	Nrf2-responsive cytoprotective enzyme	PM2.5, PM10	Biomarker of oxidative stress
Nrf2	Redox regulator	Activated	Induces antioxidant and detoxification genes	PM, metals	Adaptive antioxidant response
MDA (Malondialdehyde)	Lipid peroxidation	Increased	Membrane oxidative damage	PM, O_3_	Indicator of lipid oxidation
Lipid Peroxides	Oxidative damage	Increased	Membrane injury	PM	Oxidative membrane damage
Protein Carbonyls	Protein oxidation	Increased	Oxidative protein modification	PM, O_3_	Protein oxidation biomarker
8-OHdG	DNA oxidation	Increased	Oxidative DNA damage	PM2.5, PAHs	DNA damage biomarker
IL-1β	Proinflammatory cytokine	Increased	Promotes acute inflammation	PM, PAHs	NF-κB/NLRP3 activation
IL-6	Proinflammatory cytokine	Increased	Systemic inflammatory mediator	PM, NO_2_, O_3_	Predictor of chronic inflammation
IL-18	Proinflammatory cytokine	Increased	Released after inflammasome activation	PM	Marker of NLRP3 activation
TNF-α	Proinflammatory cytokine	Increased	Amplifies inflammatory cascade	PM, NO_2_	Systemic inflammatory biomarker
RANTES (CCL5)	Chemokine	Increased	Leukocyte recruitment	PM	Early airway inflammation
NF-κB	Transcription factor	Activated	Regulates inflammatory gene expression	PM, PAHs	Master inflammatory regulator
MAPKs (ERK, JNK, p38)	Signaling proteins	Activated	Stress-responsive inflammatory signaling	PM, NO_2_	Mediates cytokine production
NLRP3 Inflammasome	Innate immune sensor	Activated	Caspase-1 activation and cytokine maturation	PM	Inflammasome-mediated inflammation
Caspase-1	Protease	Activated	Cleaves IL-1β and IL-18	PM	Marker of inflammasome activation
COX-2	Inflammatory enzyme	Increased	Prostaglandin synthesis	PM	Indicator of inflammatory signaling
NO (Nitric Oxide)	Inflammatory mediator	Increased	Nitrosative stress and immune activation	PM, NO_2_	Airway inflammatory marker
Mitochondrial DNA release	Mitochondrial damage	Increased	DAMP-mediated inflammation	PM2.5	Mitochondrial dysfunction biomarker
GRP78, ATF4, XBP1	ER stress markers	Increased	Unfolded protein response activation	PM, cigarette smoke	Biomarkers of ER stress

**Table 2:** Associations between major air pollutants, oxidative stress markers, molecular pathways, and human health outcomes.

Pollutant	Oxidative Stress Marker	Molecular Pathway	Associated Outcome	References
PM2.5	ROS, MDA, 8-OHdG, GSH	Nrf2, NF-κB, NLRP3	COPD, cardiovascular effects	[28]
O_3_	ROS, GSH, MDA	Nrf2, NF-κB, MAPK	Airway inflammation	[29]
NO_2_	ROS, inflammatory cytokines	NF-κB, MAPK	Airway and vascular effects	[30]
PAHs	8-OHdG, ROS	AhR, oxidative stress pathways	DNA damage and cancer-related effects	[31]
SO_2_	ROS, lipid oxidation	Inflammatory signaling	Airway inflammation	[32]
VOCs	ROS, oxidative damage	Oxidative/inflammatory pathways	Neurotoxicity and respiratory effects	[33]

## Data Availability

No new data were created or analyzed in this study. Data sharing is not applicable to this article.
